# An institutional study: Does Body Mass Index influence surgical approach, surgical morbidities, and outcomes in endometrial cancer patients?

**DOI:** 10.52054/FVVO.15.3.081

**Published:** 2023-09-24

**Authors:** P Español, A Luzarraga, N Teixeira, C Soler, R Luna-Guibourg, R Rovira

**Affiliations:** Department of Obstetrics and Gynecology. Hospital de la Santa Creu i Sant Pau, C/ Sant Quintí 89, 08041, Barcelona, Spain; Gynecology and Oncology Peritoneal Group, Institut d’Investigacions Biomèdiques Sant Pau, Hospital de la Santa Creu i Sant Pau, C/ Sant Quintí, 77-79, 08041, Barcelona, Spain

**Keywords:** Endometrial cancer, laparoscopy, obesity, body mass index

## Abstract

**Background:**

Endometrial Cancer (EC), the most common genital tract malignancy in women, is recognised to be associated with a high Body Mass Index (BMI).

**Objective:**

The aim of the study was to evaluate the impact of obesity on intra and post-operative morbidity for patients treated for EC.

**Materials and Methods:**

This was a retrospective observational study including patients with EC that were surgically treated at Hospital de la Santa Creu i Sant Pau during nine consecutive years. The patients were divided in groups according to BMI: <30 Kg/m^2^, ≥30-<40 Kg/m^2^ and ≥40 Kg/m^2^. Demographic and pathological characteristics, surgical outcomes, perioperative complications and long-term outcomes were recorded.

**Results:**

The study included 290 patients; 164 patients with BMI <30 Kg/m^2^ (56.5%), 107 patients with ≥30-<40 Kg/m^2^ 36.9%) and 19 patients with ≥40 Kg/m^2^(6.65%). Patients with BMI ≥40Kg/m^2^ were younger, presented a higher percentage of endometrioid histology (84.2%, p<0.01), well-differentiated tumours (73.7%, p<0.01) and were more frequently in the initial stages at diagnosis (94.7%) compared to the other groups. A significant percentage of the patients were operated on laparoscopically (88.7%, 88.8%, 94.7% respectively). No significant differences were found in the evaluation of the surgical outcomes. The results relative to complications showed an overall tendency toward increase in the ≥40 Kg/m^2^ BMI group but no statistical differences were identified among the groups in terms of complications or long-term outcomes.

**Conclusions:**

There was a rising trend towards increased complications with increasing BMI in the study population, however, this was found not to be statistically significant. Therefore, the optimisation of co- morbidities and the adaptation of surgical treatment is important for the management of obese patients with endometrial cancer.

**What is new?:**

The study allows the comparison between groups with different BMI in patients with endometrial cancer. Different surgical outcomes, intra operative, early, and late complications are clearly identified, and survival outcomes are also investigated in our study.

## Introduction

Endometrial cancer (EC) is a major health problem globally as one of the most important risk factors, obesity, is steadily increasing. Furthermore, EC is the most common gynaecological cancer in developed countries and the second most prevalent for mortality after ovarian cancer (Siegel et al., 2018; Bittoni et al., 2013).

Obesity is defined by using a Body Mass Index (BMI) cutoff point of ≥30Kg/m^2^. Currently, 22% of the female population in Spain between the ages of 64 and 84 classify as obese. The biological mechanism that relates excess endogenous oestrogen in peripheral fat with an increased risk for EC is well established (Onstad et al., 2016; McCullough et al., 2008; Shaw et al., 2016). Obese women have a 2 to 5-fold higher incidence of EC, meanwhile an increase in the BMI by 5Kg/m^2^ raises the risk of EC by an RR of 1.59 (95% CI 1.50-1.68) (Renehan et al., 2008). Obesity is not the only the risk factors for developing the disease, but also increases the associated disease mortality. Up to some 80% of deaths in patients with stage I-II EC are secondary to other disease-independent causes (Robbins et al., 2013), with cardiovascular disease being the leading cause of death reported (Ward et al., 2012; Secord et al., 2016).

80% of patients with EC are diagnosed in the early stages with a 5-year survival rate of more than 95%. However, 20% are diagnosed in the advanced stage, which reduces the 5-year survival rate to below 70% (Concin et al., 2021). The treatment of EC depends on the stage and is carried out in accordance with the clinical guidelines (Concin et al., 2021; Oncoguía SEGO, 2017). The optimum standard of care to ensure proper management of EC is surgery. Obesity potentially increases the risk of perioperative complications. It can result in longer hospital stay, delay in adjuvant treatment and generation of additional treatment costs (Orekoya et al., 2016). The BMI cutoff at which the complication rate in EC increases significantly is a BMI ≥40Kg/m^2^ (Jan et al., 2014).

Minimally invasive surgery has been shown to improve the peri-operative results without affecting the oncological outcomes when compared to traditional open laparotomy. Therefore, it constitutes the current preferred approach to the management of EC (Concin et al., 2021; Ju et al., 2009; Zullo et al., 2012; Walker et al., 2009). A recent review of the Cochrane Library concluded that the laparoscopic pathway is associated with lower morbidity and shorter hospital stay in early- stage EC patients (Galaal et al., 2018). On the other hand, issues such as the increased surgical time, the ventilatory difficulty linked to the Trendelenburg position or the rate of conversion to laparotomy remain present in the analysis of the minimally invasive laparoscopic approach. The surgical management of obese patients is a challenge. There are numerous publications in the literature that analyse the impact of the BMI on the surgical approach and on the morbidity of EC patients, mostly in North American. It is attributable to the high rates of obesity in the region’s population (Suidan et al., 2017; Gambacorti-Passerini et al., 2019; Bouwman et al., 2015; Ucella el at., 2016).

Obtaining epidemiological data and knowledge of the surgical outcomes associated with the increase in the BMI in our population are important factors that aid counselling patients and optimising EC management. The aim of this study was to evaluate the impact of obesity on surgical outcomes and assess intra-operative and post-operative morbidity in patients with EC at our institution. An additional second objective was to assess the long-term outcomes related with the BMI.

## Materials and Methods

This was a retrospective observational study that included patients with a histological diagnosis of EC that were consecutively surgically treated at Hospital de la Santa Creu i Sant Pau in Barcelona between 2010 and 2019.

All patients were evaluated by the institutional Gynaecology-Oncology Committee and the therapeutic decision, which included the adjuvant treatment and follow-up, was based on existing institutional protocols for EC (Concin et al., 2021; Oncoguía SEGO, 2017). Participation did not imply any changes in standard care and all patients signed an informed consent.

Initial surgical staging included a total hysterectomy with a bilateral salpingo-oophorectomy. Pelvic and paraaortic lymphadenectomy was indicated in cases of myometrial infiltration of ≥50%, grade 3 endometrioid endometrial tumour or type 2 EC. An omentectomy was added in the latter subtypes. The same team of four experienced surgeons from the Gynaecology Oncology Unit of our institution performed all surgical procedures in accordance with the International Federation of Gynecology and Obstetrics (FIGO) guidelines (Pecorelli, 2009).

The exclusion criteria covered patients with stage IV disease (FIGO Classification) or with insufficient clinical/pathological data. The data of 324 patients with EC were retrospectively collected based on hospital records. It was then introduced into the online Clinapsis database by two authors of this article. At the end of the process, 290 were selected for the present study.

The variables recorded included the demographic and pathological characteristics, surgical outcomes and perioperative complications and long-term outcomes. Complications were classified as intraoperative, 30-day postoperative (early) and after 30-day postoperative (late) complications. At the same time, they were categorised as anaesthetic, urologic, intestinal, vascular, respiratory- cardiac, wound, thromboembolic or lymphatic complications. The Clavien-Dindo classification (Radosa et al., 2014) was used to determine its severity. For the purposes of the study, patients were divided into three groups according to their BMI: BMI <30 Kg/m^2^, BMI ≥30 - <40 Kg/m^2^ and BMI ≥40 Kg/m^2^.

The study was approved by the appropriate Ethics Committee of our institution with the code: EC/21/187/6429. Written informed consent for the operation and for their data to be used in clinical research was obtained from all the patients.

### Statistics

Data was exported from the Clinapsis platform into a Microsoft Excel format for processing with the IBM-SPSS V26 Statistics software. The student’s t-Test or the ANOVA test was applied for continuous variables, depending on their distribution. The Pearson Chi-square test or Fisher’s exact test was used to calculate categorical variables. The survival analysis was performed with the Kaplan Meier method while comparisons of survival were made using the log-rank test. Survival outcomes were calculated from the date of final treatment of the primary disease, surgery, or end of adjuvant chemotherapy up to the date of recurrence, the last visit or death. Values of p<0.05 were considered significant differences.

## Results

A total of 290 EC affected patients were surgically staged and included in the study. The average BMI was 29.88Kg/m^2^ (SD 6.76, 16.96-59.52 Kg / m^2^). There were 164 patients (56.5%) with a BMI <30Kg/m^2^, 107 patients (36.9%) with a BMI ≥30 - <40Kg/m^2^ and 19 patients (6.65%) with a BMI ≥40Kg/m^2^.

The baseline demographic and pathological analysis are included in Table I. Due to obesity characteristics, a predictable correlation between the increase in BMI and ASA score was expected (p<0.01), unlike for the Karnofsky Index. The average age was of 74 years for the overall cohort, while it was 5 years less in the BMI ≥40Kg/m^2^ group. The majority of patients presented with stage I disease, 75%, 85% and 94,7% respectively by BMI group. Moreover, patients with a BMI ≥40Kg/m^2^ were more likely to present a higher percentage of endometrioid histology (84.2%, p<0.01) and of differentiated tumours (73.7%, p<0.01), while patients with BMI <30Kg/m^2^ were more likely to present a higher percentage of a non-endometroid histology (37.2%, p<0.01), high grade 3 tumours (53%, p<0.01) and FIGO Stage III-IV (25%, p<0.01).

**Table I:** Baseline demographic and pathological characteristics.

		BMI <30 Kg/m^2^	BMI ≥30 - <40 Kg/m^2^	BMI ≥40 Kg/m^2^	p value
N		164	107	19	
Age, years (mean, SD)		74.5 (14.2)	74.9 (12.2)	69.6 (11.9)	0.27
ASA score (N, %)					<0.01
	Score ≤2	95 (57.9)	45 (42.1)	5 (26.3)	
	Score ≥3	69 (42.1)	62 (57.9)	14 (73.7)	
Karnofsky Index (N, %)					0.68
	Score ≤80	55 (33.5)	41 (38.3)	6 (31.6)	
	Score ≥90	109 (66.5)	66 (61.7)	13 (68.4)	
Histological type (N, %)					<0.01
	Endometrioid	103 (62.8)	90 (84.1)	16 (84.2)	
	Non-endometroid	61 (37.2)	17 (15.9)	3 (15.8)	
Tumor size, cm (mean, SD)		3.96 (2.8)	3.67 (2.5)	3.24 (1.6)	0.53
Histological Grade (N, %)					<0.01
	Grade I - II	77 (47)	68 (63.6)	14 (73.7)	
	Grade III	87 (53)	39 (36.4)	5 (26.3)	
FIGO Stage (N, %)					0.31
	Initial stage (FIGO I-II)	123 (75)	91 (85)	18 (94.7)	
	Advanced stage (FIGO III-IV)	41 (25)	16 (15)	1 (5.3)	

*FSFI Score <26.55 is consistent with female sexual dysfunction.

**Figure 1:**
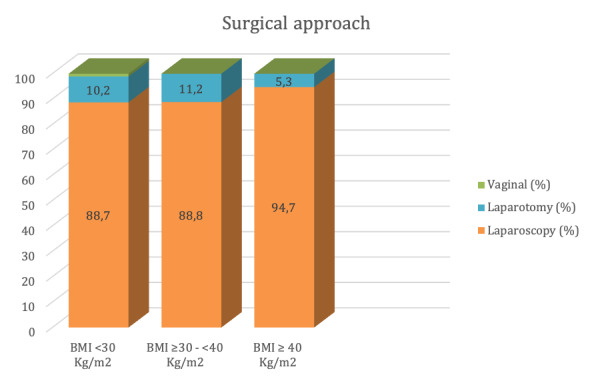
Surgical approach related to BMI groups.

Surgical outcomes are shown in Table II. After a complete evaluation, no significant differences were reported across the three BMI groups. A significant percentage of the patients were operated by laparoscopy (overall 88.29%), including patients with a BMI ≥40Kg/m^2^ (Figure 1). Among non- laparoscopy reasons, data distribution included 3.1% severe intra-abdominal adhesions, 5.8% large tumours, 0.7% serious medical comorbidities and 2.1% of other reasons. The chart notes two cases of a vaginal approach, both included in the BMI <30Kg/m^2^ group. One was the unexpected finding of an endometrioid neoplasia following prolapse surgery. The basic preoperative study based on anamnesis and vaginal ultrasound had not identified the neoplasm. The final stage was IA. With no associated risk factors, the surgery was completed with a laparoscopic bilateral adnexectomy. The other was a FIGO stage IA, grade 1 neoplasia, that presented in a 90-year-old patient with uterine prolapse and a severe cardiac pathology (severe ischemic mitral regurgitation with associated pulmonary hypertension). The Gynecology-Oncology Committee in accordance with the patient decided to perform a hysterectomy and bilateral adnexectomy though the vaginal approach.

**Table II:** Surgical outcomes.

		BMI <30 Kg/m^2^	BMI ≥30 - <40 Kg/m^2^	BMI ≥40 Kg/m^2^	p value
N		164	107	19	
Surgical approach (N, %)					0.46
	Laparoscopy	145 (88.7)	95 (88.8)	18 (94.7)	
	Laparotomy	17 (10.2)	12 (11.2)	1 (5.3)	
	Vaginal	2 (1.21)	(0)	0 (0)	
Lymphadenectomy (N, %)					
	Pelvic	74 (45,1)	47 (43.9)	4 (21.1)	0.79
	Paraaortic	69 (42,1)	36 (33.6)	4 (21.1)	0.23
Lymph nodes removed (median, IQR p25-p75)					
	Pelvic	12 (8-17)	13 (9-17)	10 (8-13)	0.27
	Paraaortic	10 (5-14)	8 (3-14)	9 (2-19)	0.53
Operative time, minutes (mean, SD)		151.0 (109.4)	154.2 (92.6)	158.7 (100.4)	0.45
Blood loss, ml (mean, SD)		179.2 (243.1)	199.2 (354.5)	234.2 (459.7)	0.70
Surgical re-exploration (N, %)		7 (4.3)	3 (2.8)	1 (5.3)	0.77
Conversion to laparotomy (N, %)		3 (1.8)	5 (4.7)	2 (10.5)	0.37
Hospital stays, days (mean, SD)		4.94 (4.7)	5.66 (13.5)	4.26 (4.9)	0.73
Time to chemotherapy, days (median, IQR p25-p75)		46.5 (34-81)	36.5 (32-65)	45.5 (42-80)	0.75

Regarding operative time, blood loss and surgical re-exploration, the data showed a progressive increase in all domains that correlated with a higher BMI. Surgical re-exploration reasons were three intestinal perforations, two ureteral/vesical lesions, one reintervention for hemoperitoneum, two lymphocele drainages and three surgical wound revision. In reference to conversion to laparotomy, the percentage of affected patients also increased proportionately to BMI. The specific data were two vascular lesions with major bleeding (external iliac vein and splenic vessels), one intestinal perforation at trocar placement, one patient intolerance to pneumoperitoneum in the BMI <30 Kg/m 2 group and six technical difficulties as voluminous uterus for associated fibromas or severe adherences.

As for the patients who underwent pelvic (p=0.79) and paraaortic (p=0.23) lymphadenectomy, the corresponding lymph nodes obtained (p=0.27; p=0.53), showed no significant differences when the three BMI groups were compared. However, the percentage associated with lymphadenectomy is notably lower in the BMI ≥40Kg/m2 group at 21.1%. For that reason, we also analysed the percentage of patients’ that could not have complete staging surgeries because of obesity or associated co-morbidities reasons. Results according to BMI groups were 13.9% (13/93 indicated), 13.2% (7/53 indicated) and 42% (3/7 indicated) (p=0.08). No significant difference was noted with regards to hospital stay (p=0.73) and time to start the chemotherapy (p=0.75).

The three categories of complications revealed an overall trend towards an increased proportion in the ≥40Kg/m2 BMI group: as shown by the data in Table III. Despite this, no statistical differences were identified. As for the intra-operative complications (26 in total, overall incidence 9.45%), the most reported were vascular injuries occurring in 10 patients among all the groups and including lesions in both internal and external iliac veins as well as inferior cava vein and epigastric artery. They were followed by urological lesions, bowel lesions, nerve lesions and finally two anaesthetic complications, one patient unable to tolerate pneumoperitoneum and another an atrioventricular block.

**Table III:** Intraoperative and postoperative complications.

	BMI <30 Kg/m^2^	BMI ≥30 - <40 Kg/m^2^	BMI ≥40 Kg/m^2^	p-value
N	164	107	19	
Intraoperative complications (N, %)	16 (10.5) 8 Vascular injuries 1 neural injury 4 Urologic injuries 2 Intestinal injuries 1 Anaesthetic complication	8 (7.4) 1 Vascular injury 2 neural injuries 2 Urologic injuries 2 Intestinal injuries 1 Anaesthetic complication	2 (10.6) 1 Vascular injury 1 Intestinal injury	0.65
30-day postoperative (Early) complications (N, %)	21 (13.8) 3 Wound infection 3 Cardiac-Respiratory 6 Intestinal (including paralytic ileus) 8 Urologic (including urinary tract infection) 1 Lymphocele	16 (14.9) 3 Wound infection 2 Venous thrombo-embolism 6 Cardiac-Respiratory 1 Hematoma - Haemorrhage 1 Intestinal (including paralytic ileus) 1 Urologic (including urinary tract infection) 2 Lymphocele	5 (26.3) 1 Wound infection 1 Venous thrombo-embolism 1 Cardiac-Respiratory 2 Intestinal (including paralytic ileus)	0.18
After 30-day postoperative (Late) complications (N, %)	3 (2) 1 Wound infection 1 Urologic (including urinary tract infection) 1 Lymphocele	3 (2.8) 1 Wound infection 1 Urologic (including urinary tract infection) 1 Lymphocele	1 (5.3) 1 Wound infection	0.57
Clavien-Dindo complications (Early) (N, %)				
1-2	15 (9.1)	12 (11.2)	4 (21.1)	0.11
≥3	6 (3.6)	4 (3.7)	1 (5.2)	

Early complications were the most interesting subgroup; there was a clear correlation with the increase in the BMI groups (13.8%, 14.9%, and 26.3%, respectively). Surgical wound infections were listed in the three groups; cardiac-respiratory events consisting of congestive heart failure or respiratory infection were also remarkable; intestinal complications ranging from paralytic ileus to perforations and finally two and one venous thrombo-embolism were identified in the BMI ≥30 - <40 and ≥40 Kg/m2 group respectively. Despite this, there were no statistical differences and Clavien-Dindo classification grade ≥ III included only 3,6%, 3,7% and 5,2% of patients by BMI group. The analysis of late complications showed a lower percentage when compared to the other complication groups.

**Figure 2:**
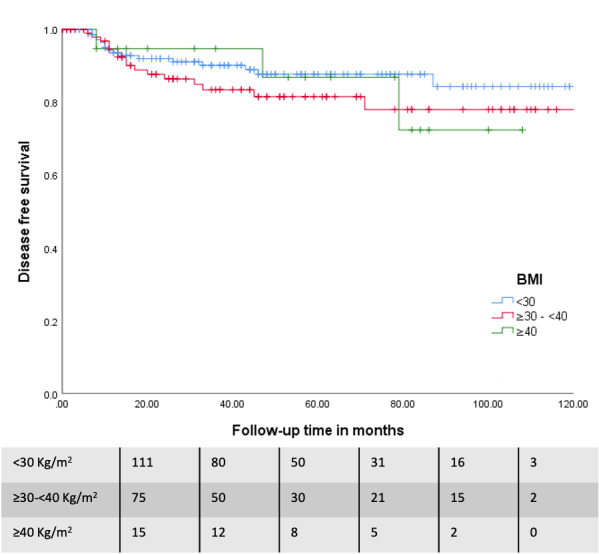
Disease free survival (DFS) according to BMI groups in endometrial cancer patient’s population.

**Figure 3:**
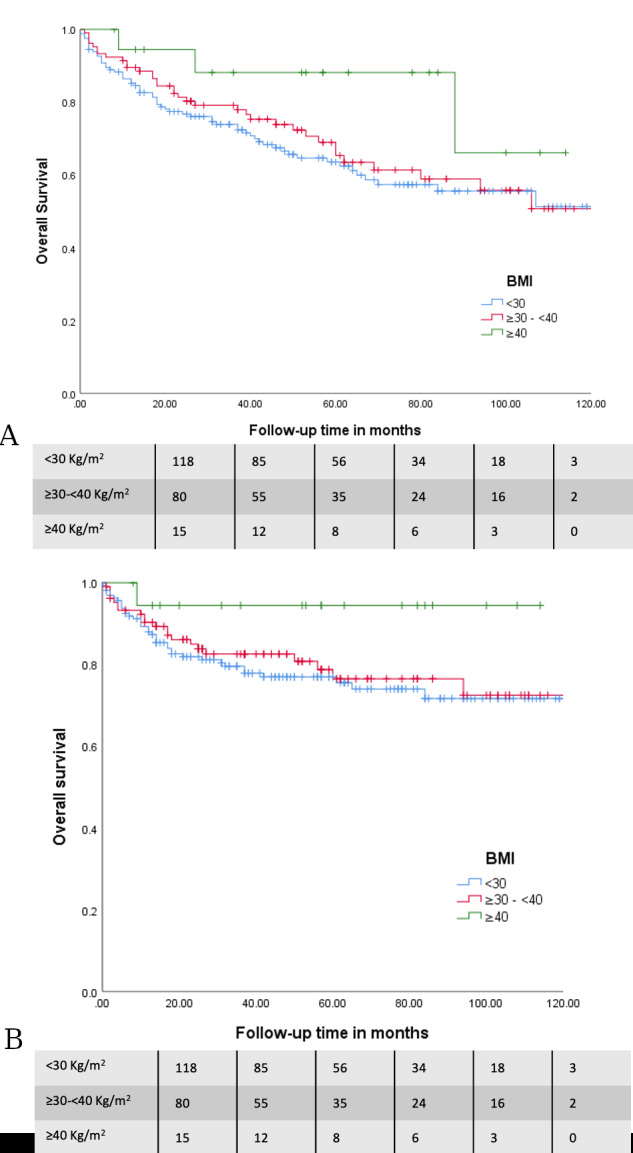
Overall survival (OS) according to BMI defined groups. All-cause mortality is shown in figure A and Endometrial cancer related mortality is exposed in figure B.

The mean follow-up of the cohort was 47.2 months (SD 35.7) (3.9 years). No significant differences were found in survival outcomes between the three groups. A trend towards an increased recurrence rate was observed with increasing BMI (recurrence rates of 9.8%, 15% and 15.8% for the three BMI classification groups respectively; p=0.38), which is probably secondary to the oestrogen physio-pathology characteristic of women with obesity. Similar results were observed for recurrence-free survival (RFS), with a nonsignificant trend towards shorter RFS with increasing BMI (126.7 +/- 4.1; 107.3 +/- 5.1; 93.89 +/- 7.3 months respectively; p=0.6). Overall Survival (OS) was calculated in two modalities: all-cause mortality (37%, 33,6%, 15.8%, respectively; p=0.15) and EC mortality (22.6%, 19.6%, 5.3%, respectively; p=0.20). Unexpectedly, mean survival time tended to be higher in patients with a BMI ≥40Kg/m 2 (overall survival of 89.2 +/-5.5; 89.9 +/- 5.5 and 96.4 +/- 8.5 months respectively; p=0.26, and EC survival of 110.9 +/- 4.9; 103.1 +/- 5,1 and 107.9 +/- 5,6 months respectively; p=0.25). These results are presented in Kaplan-Meier survival curves (Figure 2 and Figure 3).

Finally, the first month mortality for all-cause pathology, associated with surgical outcomes and complications, also showed no significant differences between the groups (Corresponding to BMI groups: 1,2%, 1.8%, 5.5%, p=0,140).

## Discussion

Laparoscopy is currently a safe and effective approach in patients with obesity (Suidan et al., 2017; Gambacorti-Passerini et al, 2019; Ucella et al., 2016; Simpson et al., 2020). This fact is reflected in the results of our study where, considering the challenge that operating on high BMI patients associating EC represents (Bouwman et al., 2015; Gunderson et al., 2014), 88% of the patients underwent a laparoscopic procedure. Several points need to be considered in the detailed analysis of the impact of BMI on the surgical management of our patients.

Regarding the entire analysis, the heterogeneity in the number of patients in each subgroup, especially in the BMI group ≥40Kg/m2, is due to its low prevalence in our population. Only 19 cases were included. The justification for the creation of this BMI group compared to the alternative of separating patients into only under and over BMI 30Kg/m2., is that other studies dealing with this topic present interesting results if compared to the BMI ≥40Kg/m2 group (Gunderson et al., 2014).

With the baseline demographic characteristics analysis, a global assessment of the operated patient with EC was made. It mainly highlighted that our population had an average age of 74 years old, which differs from that of the United States and other populations studied, with an average age of 61 years old (Simpson et al., 2020)

One of the controversial aspects around the use of minimally invasive surgery in patients with obesity is the increase in surgical time. Some studies describe prolongation of surgery while other studies do not identify significant differences (Walker et al., 2009; Radosa et al., 2014). No differences were observed in this study either. Upon analysing the conversion rate from the initial laparoscopic route to laparotomy, our data showed an increase in it in patients with BMI ≥30Kg/m2. Those results agree with the data in the literature (Bouwman et al., 2015; Ucella el at., 2016). However, in the extreme obese group, only two patients out of nineteen had to be converted to laparotomy.

Previously, some studies have documented the significant impact of obesity on the decision to perform lymphadenectomy (Ucella el at., 2016; Gunderson et al., 2014). In our results, there is a noticeable trend toward fewer lymphadenectomies being performed the more obese the patient is, although no significant differences were found between the BMI three groups in terms of staging surgery or in the number of lymph nodes removed. The histological and the diagnosis stage characteristics of highest IMC women in this study are in concordance with the need for fewer staging surgeries. Despite this, obesity is the main cause to not perform a complete staging surgery in 42% of indicated cases of BMI group ≥40Kg/m2. The low number of patients included in the group could also influence these results. As the age is also a factor related to co-morbidities that affects the decision to perform a lymphadenectomy, a study encompassing older age of patients have been recently published in our institution (Luzarraga- Aznar et al., 2022). As the last item related to staging, a current strategy that can be also useful to explore the staging at the same time to avoid complete lymphadenectomy and its morbidity in selected patients is a sentinel node study, already established in some institutional protocols (Concin et al., 2021; Oncoguía SEGO, 2017). This can be an option for all patients, especially in case of clinically severe obese patients.

Relative to complications, there were no statistically significant differences for the three BMI groups in any of the complication subtypes analysed (intraoperative, early, late), which is not in agreement with most published studies (Suidan et al., 2017; Bouwman et al., 2015; Ucella et al., 2016; Gunderson et al., 2014). In this aspect, the laparoscopic approach used in more than 88% of patients can be the cause. Laparoscopy markedly decreases complications, as well as costs, when compared to laparotomy, being even more relevant in high BMI patients (Gambacorti-Passerini et al., 2019). Most complications were identified in the early complications group. Co-existing comorbidities, thrombotic events and surgical wound infection are highlighted as more common in the literature (Bouwman et al., 2015; Ucella et al., 2016; Simpson et al.,2020). In our case, there was a marked increase in the complications rate in extreme obese patients.

Numerous studies suggest that laparoscopy is beneficial in addressing endometrial cancer in the patients with obesity (Gambacorti-Passerini et al., 2019; Bouwman et al., 2015; Ucella et al., 2016; Simpson et al., 2020; Tinelli et al., 2014) The associated cost reduction should also be assessed along with the clinical benefits (Suidan et al., 2017). For our population, data is lacking. For this reason, the present study was designed knowing the high percentage of patients operated on in our centre with a minimally invasive approach over the past ten years. As the results show, there are some points that leave room for improvement, especially in the peri-surgical period, to reduce the morbidity that surgery represents. In this respect, programs such as pre-rehabilitation added to ‘Enhanced Recovery After Surgery’ (ERAS) (Nelson et al., 2019) are associated with lower peri-operative morbidity. Established in our centre since 2019, Pre-rehabilitation and ERAS program promotes pre- and intra-surgical optimisation of both the patient and their co-morbidities in three fundamental aspects: the psychological, functional, and metabolic. This is also true for optimal post- surgical management. This programme highlights the importance of preparation prior to surgery with the aim of anticipating the problems that may occur in patients with obesity.

The impact of obesity on recurrence or disease- free survival in patients with EC is complex due to the multiple factors involved. No differences in terms of recurrence and survival between the groups in this study were seen. However, a trend towards greater global survival in patients with BMI ≥40Kg/m2 was observed. A hypothesis to explain the survival results might be the 5-year mean-age difference of these patients compared to the other subgroups as well as the histological characteristics of their tumour. We must tread with caution considering the small sample size of this subgroup. Therefore, we would probably be able to demonstrate a decrease in survival like the related literature describes if we had a longer follow-up and more patients to study (Secord et al., 2016). The changes in the curve that are shown based on the division of cause of the death into “all the causes” vs “by EC” must be highlighted. Again, there is agreement with the data in the literature (Ward et al., 2012; Secord et al., 2016). Furthermore, the results from a French multi-centre study support the importance of not under treating patients with obesity and emphasises the need to complete the surgical staging and adapt adjuvant treatment in this subgroup (Canlorbe et al., 2015). Survival in the first month after surgery for all causes does not show significant differences between the groups, which is similar to other studies (Mahdi et al., 2015).

The limitations of this study are the retrospective nature of the data, the overall small sample numbers, and the difference in groups size, especially for the BMI ≥40Kg/m 2 group. Specific data about medical co-morbidities was not available and this also represents another study limitation. The assumptions extracted in this study are presented prior to confirmation by controlled and randomised studies. However, it is still useful to have the internal centre data to detect trends in our population and identify modifiable points in daily clinical practice.

## Conclusions

According to the analysis of the results in our population, BMI does not significantly increase the peri-operative complications nor differentiate the principal surgical outcomes of the patients with endometrial neoplasia. Even though a tendency to increased early and late complications is identified in patients with a BMI ≥30 Kg/m2, it is more marked in a BMI >40 Kg/m2. Therefore, the optimisation of co-morbidities and surgical treatment based on minimally invasive techniques is very relevant for the management of obese patients with endometrial cancer.
